# Dual Energy Spectral CT Imaging in the assessment of Gastric Cancer and cell proliferation: A Preliminary Study

**DOI:** 10.1038/s41598-018-35712-w

**Published:** 2018-12-04

**Authors:** Sai-Ming Cheng, Wei Ling, Jiong Zhu, Jian-Rong Xu, Lian-Ming Wu, Hong-Xia Gong

**Affiliations:** 1 Department of Radiology, Renji Hospital, School of Medicine, Shanghai Jiaotong University, Shanghai, 200127 China; 2 Department of Gastrointestinal Surgery, Renji Hospital, School of Medicine, Shanghai Jiaotong University, Shanghai, 200127 China

## Abstract

Gastric cancer is one of the main diseases leading to cancer-related death. The recently introduced dual-energy spectral CT (DEsCT), allows to obtain many quantitative measurements from iodine-based material decomposition (MD) images, which contribute to improve the accuracy of staging of GC comparing to multidetector spiral CT. And Ki-67 is a well-recognized nuclear antigen-specific biomarker reflecting cellular proliferation for estimating growth fractions of various tumor types. In the present study we analyzed the features of quantitative measurements (the curve slope (λ_HU_), IC, normalized iodine concentrations (NIC)) obtained from DEsCT and levels of Ki-67 protein expression. We demonstrated that the values between advanced gastric cancer (AGC) and early gastric cancer (EGC) were significantly different both in venous phase (VP) and delayed phase (DP). The values of different level of Ki-67 expression grade were significantly different both in VP and DP. The rank correlation analysis between Ki-67 grade and IC, NIC and λ_HU_ values showed significantly positive correlation in VP and DP. These results suggested that quantitative parameters (IC, NIC and λ_HU_) in dual-energy CT imaging can be used to differentiate EGC from AGC, and have significantly positive correlation with Ki-67 antigen expression levels in gastric cancer for indicating tumor cellular proliferation.

## Introduction

Gastric cancer is one of the main diseases leading to cancer-related death with estimated 951,600 new cases and 723,100 deaths in 2012, although a decreasing trend has been observed in stomach cancer incidence and mortality rates worldwide^1^. The stage of cancer is the most decisive factor for determining treatment strategies and for long-term prognosis^2^. Early gastric cancer (EGC), defined as gastric cancer in which tumor invasion is confined to the mucosa or submucosa (T1 cancer), no matter of lymph node metastasis according to the Japanese classification^3^, is usually treated with endoscopic resection and has a relatively good 5-year survival rate. Meanwhile, advanced gastric cancer (AGC) is more often treated by gastrectomy with different lymphadenectomy along with a poorer outcome^2,4,5^.

Modern imaging technology such as conventional barium enema, air-contrast radiography is often utilized to detect and noninvasively stage gastrointestinal diseases. But computed Tomography (CT) scanner, especially multidetector spiral CT (MDCT) with combined water and air inflation of stomach, is currently the most common and effective method to stage gastric cancer (GC), including assessing locoregional tumor invasion and discovering adjacent and distant metastases^6–10^. The recently introduced dual-energy spectral CT (DEsCT), allows medical researchers to obtain many quantitative measurements, such as iodine concentration from iodine-based material decomposition (MD) images, which contribute to improve the accuracy of staging of GC comparing to MDCT, and help to discriminate differentiated from undifferentiated stomach cancer, and metastatic from non-metastatic lymph nodes^11–15^. However, few previous studies have focused on differentiating EGC from AGC with this advanced tool. Therefore, one of the objectives of the present study was to identify whether DEsCT imaging could reveal significant differences between EGC and AGC with the application of quantitative iodine concentration measurements from MD images during the arterial phase (AP), venous phase (VP) and delayed phase (DP).

Another objective was to explore the potential of DEsCT measurements as prognostic indicators by establishing probable correlation with Ki-67 protein expression in gastric cancers. Ki-67 is a well-recognized nuclear antigen-specific biomarker reflecting cellular proliferation for estimating growth fractions of various tumor types^16^. Although it is still inconclusive, expression of Ki-67 antigen is considered to be associated with clinicopathological characteristics of gastric adenocarcinoma such as size of tumor, depth of invasive, grade of histology, metastasis in regional lymph nodes, clinical stage and prognosis^17–21^. Thus, it might be meaningful to study the pathologic behavior of gastric cancers from the perspective of medical imaging parameters by comparing DEsCT measurements of lesions with different Ki-67 antigen levels.

From the two above-mentioned aspects, the purpose of this work was to assess the feasibility and clinical value of DEsCT imaging in diagnosing gastric cancers with different Ki-67 antigen expressions.

## Results

**Figure 1:**
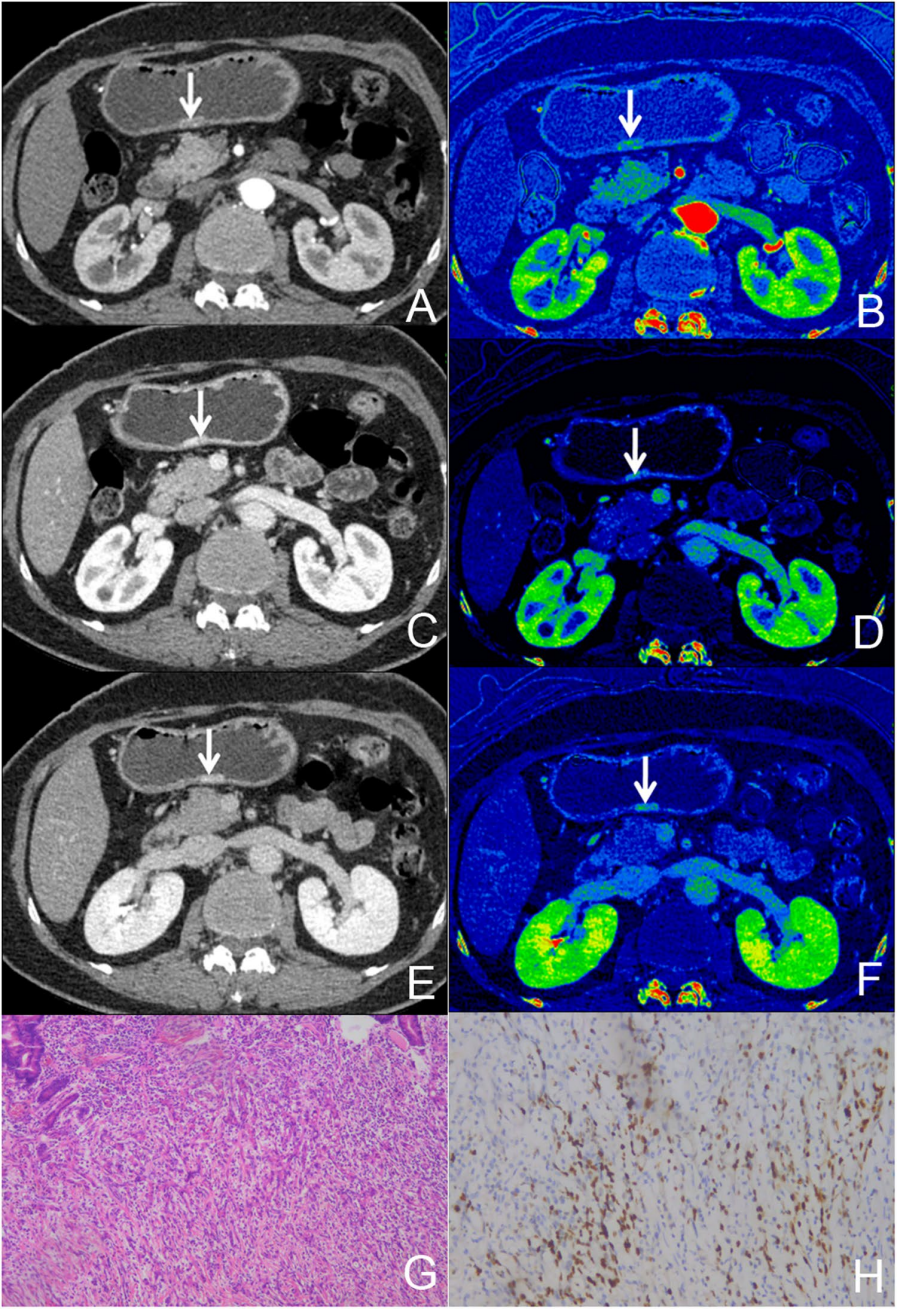
A 65-year old female with pathologically confirmed EGC with 40% of Ki-67 antigen, tumor cells invaded submucosa. (**A**,**C**,**E**) Showed monochromatic CT images of AP, VP and DP at 70 KeV. (**B**,**D**,**F**) Showed iodine-based MD images of AP, VP and DP. G showed HE-stain of cancer lesion, H showed immumohistochemical staining of Ki-67 in tumor.

**Figure 2:**
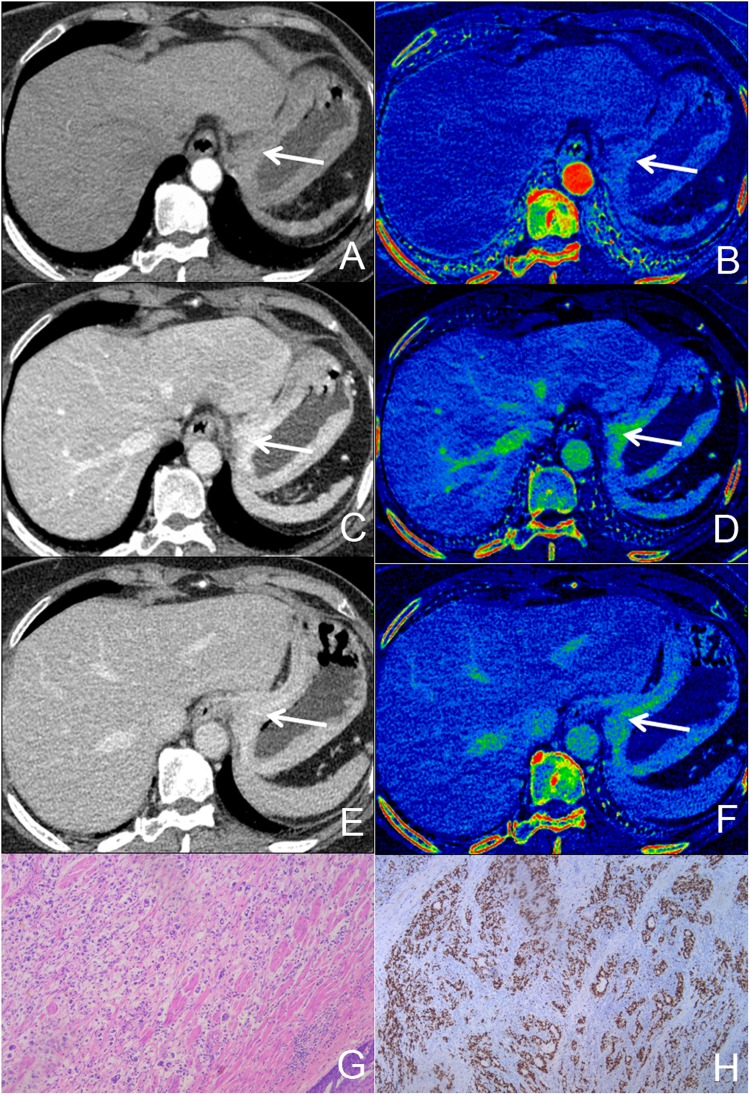
A 66-year old female with pathologically confirmed AGC with 90% of Ki-67 antigen, tumor cells invaded serosa. (**A**,**C**,**E**) Showed monochromatic CT images of AP, VP and DP at 70 KeV. (**B**,**D**,**F**) Showed iodine-based MD images of AP, VP and DP. G showed HE-stain of cancer lesion, H showed immumohistochemical staining of Ki-67 in tumor.

**Figure 3:**
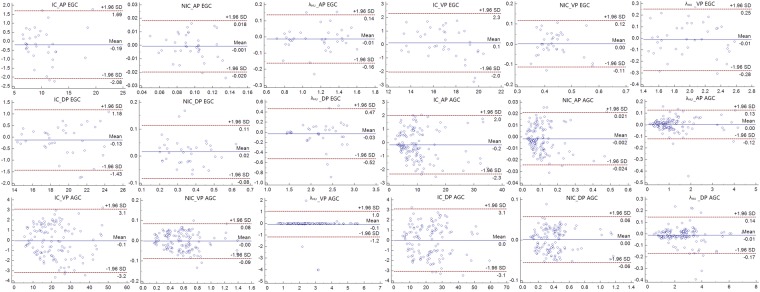
Bland-Altman plots of the IC, NIC and λHu of all participants during triple phases demonstrate good agreement within two observers.

The virtual monochromatic CT images at the 70 keV energy level, iodine-based MD images in the three phases and histopathological images of the HE-staining and immumohistochemical staining of Ki-67 of a patient with AGC and EGC are shown in Figs 1 and 2, respectively. All the measurements were expressed as mean values ± standard deviation (SD). Two independent observers performed a variability analysis for every participant. Figure 3 shows the Bland-Altman plots of the IC, NIC and λ_HU_ of all participants, demonstrating good agreement within two observers. The IC, NIC and λ_HU_ values of the EGC group and the AGC group are shown in Table 1. The values between EGC and AGC groups were significantly different both in VP (mean IC, 19.36 mg/mL ± 2.82 vs 21.25 mg/mL ± 4.91; mean NIC, 0.35 ± 0.11 vs 0.42 ± 0.12; mean λ_HU_, 2.20 ± 0.43 vs 2.67 ± 0.63; p < 0.05) and DP (mean IC, 16.89 mg/mL ± 2.07 vs 19.10 mg/mL ± 4.07; mean NIC, 0.43 ± 0.06 vs 0.58 ± 0.14; mean λ_HU_, 1.99 ± 0.35 vs 2.51 ± 0.64; p < 0.01), but not in AP (all P > 0.05).

**Table 1:** The mean value of IC, NIC and λHu values of EGC, AGC in triple phases.

	EGC	AGC	*P*
IC_AP	10.14 ± 3.24	9.71 ± 3.42	0.495
NIC_AP	0.09 ± 0.02	0.10 ± 0.03	0.120
λHu_AP	1.15 ± 0.26	1.20 ± 0.35	0.432
IC_VP	19.36 ± 2.82	21.25 ± 4.91	0.029
NIC_VP	0.35 ± 0.11	0.42 ± 0.12	0.002
λHu_VP	2.20 ± 0.43	2.67 ± 0.63	0.000
IC_DP	16.89 ± 2.07	19.10 ± 4.07	0.002
NIC_DP	0.43 ± 0.06	0.58 ± 0.14	0.000
λHu_DP	1.99 ± 0.35	2.51 ± 0.64	0.000

The spectral CT parameters for the Ki-67_L, the Ki-67_M and the Ki-67_H groups are shown in Table 2. The multiple comparisons among the Ki-67_L, i-67_M and Ki-67_H groups were significantly different both in VP (mean IC (in mg/mL), 16.08 ± 2.85, 20.79 ± 2.32 and 25.64 ± 3.66; mean NIC, 0.29 ± 0.05, 0.41 ± 0.09 and 0.51 ± 0.09; mean λ_HU_, 1.90 ± 0.28, 2.67 ± 0.39 and 3.06 ± 0.59, respectively, p < 0.01) and in DP (mean IC (in mg/mL), 15.27 ± 1.95, 18.51 ± 2.63 and 22.10.31 ± 3.84; mean NIC, 0.42 ± 0.05, 0.54 ± 0.10 and 0.68 ± 0.12; mean λ_HU_, 2.02 ± 0.38, 2.39 ± 0.38 and 2.78 ± 0.74, respectively, all p < 0.01). The results of the correlation analysis between Ki-67 grade and IC, NIC and λ_HU_ values are shown in Table 3. The Spearman rank correlation analysis demonstrated that Ki-67 grade and IC, NIC and λ_HU_ values had significantly positive correlation with correlation coefficients of 0.818, 0.753, 0.728, respectively in VP and 0.730, 0.745, 0.468, respectively in DP.

**Table 2:** The mean value of IC, NIC and λHu values of Ki-67_L group, Ki-67_M group, Ki-67_H group in triple phases.

	Ki-67_L	Ki-67_M	Ki-67_H
IC_AP	8.65 ± 3.29	10.32 ± 3.30	10.12 ± 3.39
NIC_AP	0.09 ± 0.02	0.10 ± 0.03	0.11 ± 0.03
λHu_AP	1.10 ± 0.25	1.20 ± 0.32	1.27 ± 0.40
IC_VP	16.08 ± 2.85	20.79 ± 2.32^※^	25.64 ± 3.66^*#&^
NIC_VP	0.29 ± 0.05	0.41 ± 0.09^※^	0.51 ± 0.09^*#&^
λHu_VP	1.90 ± 0.28	2.67 ± 0.39^※^	3.06 ± 0.59^*#&^
IC_DP	15.27 ± 1.95	18.51 ± 2.63^※^	22.10 ± 3.84^*#&^
NIC_DP	0.42 ± 0.05	0.54 ± 0. 10^※^	0.68 ± 0.12^*#&^
λHu_DP	2.02 ± 0.38	2.39 ± 0.53^※^	2.78 ± 0.74^*#&^

Note: values are presented with mean ± standard deviation.*Indicates difference between multiple samples. ^#^and ^※^indicate difference with Ki-67_L group. &Indicates difference with Ki-67_M group. *p* value of these comparisons is less than 0.01.

**Table 3:** The result of rank correlation analysis between Ki-67 grade and IC, NIC and λHu values in triple phases.

	r	*P*
IC_AP	0.094	0.235
NIC_AP	0.069	0.386
λHu _AP	0.099	0.598
IC_VP	0.818	0
NIC_VP	0.753	0
λHu _VP	0.728	0
IC_DP	0.730	0
NIC_DP	0.745	0
λHu _DP	0.468	0

Note: *P* value less than 0.01 is considered statistically significant.

## Discussion

Gastric cancer remains one of the dominating causes of cancer-related mortality worldwide and most prevalent cancer in Eastern Asia^5^, where more than half of the treated patients are with an early stage tumor because of nationwide screening programs^4,22,23^. With the advantage of rapid imaging and good low contrast resolution, CT examination becomes a routine method of preoperative evaluation in clinical use. Distending the gastric wall is beneficial to observe tumorous lesions during CT scans, however, it is also making gastric wall too thin to estimate the invasion depth of early stage cancer. Cancer cells often infiltrate into the deeper layer of gastric wall even in early stage, unfortunately it is hard to detect the depth of tumor invasion with MDCT owing to the limitation of the resolutions of conventional CT^24^. It hints that low contrast resolution needs to be further improved and quantitative measurement may be helpful to explore the severity of cancer cell infiltration in stomach wall, in other words, to differentiate EGC and AGC. The attenuation of matters is based mainly on the Compton effect when the X-ray energy is at above 40 keV, and this mechanism depends on energy of X-ray and attenuating material. By analyzing X-ray spectrum at two different energy levels simultaneously, DEsCT imaging allows to further acquire different virtual monochromatic datasets for improved contrast resolution and material-decomposition images for better material separation^13,25–27^. Iodine concentration in lesions is often derived from iodine-based material decomposition image to improve the detection of small lesions^28^. Thus DEsCT, with many measurable parameters, might be helpful in the evaluation of gastric cancers.

During conventional triple-phasic enhancement MDCT scans, the AP images was often utilized to detect lesion, the VP images to recognize the stomach from neighbor tissues and assess the condition of lymph node, and the DP images to facilitate the evaluation of the invasion depth of gastric wall^29,30^. In this study, we found IC, NIC and λ_HU_ of EGC were significantly lower than those of AGC in the latter two phases. It demonstrated that these measurements, reflecting different blood supply of different stage cancers, could distinguish EGC from AGC in VP and DP.

To better use dual-energy spectral CT to evaluate gastric cancer, we attempted to explore the potential biomarker function of the measurements derived from DEsCT by analyzing whether IC, NIC and λ_HU_ values were significantly correlated with the expression levels of Ki-67 antigen in tumor lesions. As far as we know, this report was a first research evaluating the relationship between parameters of DEsCT and Ki-67. In former studies, Ki-67 was often selected as a reference or combination maker to research probable role of new markers such as P53 and CD133 in clinicopathological characters of gastric cancer^17–21^. There were still contradictory opinions about the correlation between Ki-67 and clinicopathological characters such as invasive depth. However, it seemed Ki-67 is a widely believed nuclear antigen-specific biomarker linked with cellular proliferation and prognosis of gastric cancer. In our study, IC, NIC and λ_HU_ values were found significantly different among the low-, medium- and high-level Ki-67 expression groups in VP and DP, and the higher grades of Ki-67, the bigger values of these parameters. When the correlation between Ki-67 grade and IC, NIC and λ_HU_ values were further analyzed, positive correlations were found statistically significant in VP and DP. And a past study showed that the NIC values for differentiated carcinoma was significantly lower than undifferentiated carcinoma^12^. These similar phenomena might attribute to the abundant blood supplying the vigorous cell proliferation in both the high level of Ki-67 and undifferentiated carcinoma. It also deserved to be mentioned that IC values in VP were biggest in the triple-phase CT scans both in the groups of different stage and Ki-67 expression, meanwhile, correlation coefficients between Ki-67 grade and IC value were largest among all the significantly different groups, indicating that IC value during VP might be the most beneficial value during these parameters. On the other hand, NIC value may not able to offer more than IC value in spite of normalizing the iodine concentration measurement to that in aorta at the same imaging level and phase to minimize the variations resulted from different circulation status of different patients.

There are several limitations in our study. Firstly, different pathological types of gastric cancer were not considered in this investigation due to the insufficient number of EGC patients. And the stage of cancer is the most crucial factor for defining treatment strategies and affecting prognosis. Therefore, we did not further analyze the difference between specific pathological tumor types of two stage cancers in the present study. Secondly, some lesions especially in EGC was hard to draw a precise ROI because of the thin wall and small size of the lesions, value deviation cannot be absolutely eliminated when ROIs were drawn manually, thus may influence the final measurements. Thirdly, it demonstrated significantly positive correlation between Ki-67 grade and IC, NIC and λ_HU_ values based on our results, however the correlation coefficients were relatively small, resulting that Ki-67 expression level could not be directly obtained from CT spectral imaging with a robust confidence. Finially, Although the two radiologists were blinded to endoscopic results they apparently were aware of a possible pathology/prevalence thus they were dealing with a particular population found to be affected by a medical condition. This is constrain that easily can bias the results of the reviewing process by the radiologists. However, we do the analysis base on quantitative measurement and these parameters are objectively gained in the processing software. Consequently, the bias may negligible in this situation.

In summary, dual-energy spectral CT imaging provides a quantitative approach for distinguishing EGC from AGC. And based on this work, iodine concentration, slope of HU curve, especially normalized iodine concentration could be worthy parameters for evaluating expression levels of Ki-67 antigen in gastric cancer, which warrants further preoperative-postoperative radiologic-pathologic comparison imaging study.

## Materials and Methods

### Patients

This prospective study was approved by the Renji hospital institutional research ethics board and written informed consent was obtained from all study participants. We confirm that all methods were performed in accordance with the relevant guidelines and regulations. A total of 162 patients known or suspected to have stomach cancer based on the result of a previous endoscopic examination underwent multiple-phase scanning with spectral CT imaging between March 2016 and October 2017 (98 men, 64 women; age range, 32–89 years; mean age, 58.4 years). All gastric cancer patients underwent surgical treatment within two weeks after CT examination. According to the pathologic findings, this cohort included 36 cases of EGC in which tumor cell infiltration was limited to submucosa, and other 162 cases of AGC with invasion exceeded submucosa. All GC cases were also divided into three pathological groups based on the grades of Ki-67 antigen expression from immunohistochemical staining pathological diagnosis: low grade (Ki-67_L, 10% −40%, n = 45), medium grade (Ki-67_M, 50% −70%, n = 72) and high grade (Ki-67_H, 80% −100%, n = 45).

### CT Examinations

Patients were required in abrosia for 8 h and to drink 1000 ml of water before CT scans. Nonenhanced and multi-phase contrast enhanced CT examinations were performed on a high definition Discovery CT750HD (GE Healthcare, Boston, USA) scanner in a craniocaudal direction. All patients lay on the scanning table stationary in the supine position. After scout CT scanning, nonenhanced scan including the entire abdomen from the septum transversum to the pelvic floor was carried out in the conventional helical mode at a tube voltage of 120 kVp. Triple-phasic enhanced scans were then performed after patient was injected nonionic iodinated contrast agent (Iopamidol 370 mg/mL; Shanghai Bracco Sine Phamaceutical, China) with a total dose of 100–140 mL (1.8 mL per kilogram of body weight) by antecubital venous access at a rate of 3.0 mL/s followed by 50 mL saline flushing through a double-syringe power injector. The enhancement scans automatically began after the attenuation value of the internal carotid level reached triggering threshold in AP, with 35 and 70 seconds delay in VP and DP, respectively. Enhancement scans were conducted in the spectral CT mode by rapid switching of tube voltages between 80 kVp and 140 kVp with a single tube on adjacent views during a single rotation. The other scan parameters of the pre-defined protocol were as follows: tube current of 600 mA, scan field of view (SFOV) 500 mm, collimation thickness of 1.25 mm, helical pitch of 1.375, and rotation speed of 0.6 second. The CT images were reconstructed automatically with adaptive statistical iterative reconstruction algorithm (ASIR) by using GSI viewer software (GE Healthcare). Two types of images were derived from the reconstruction of DEsCT imaging for each patient: water- and iodine-based material decomposition images and a set of virtual monochromatic images at energies ranging from 40 to 140 keV.

### Data acquisition

Two independent experts in abdominal radiology specialized in gastrointestinal imaging reviewed all CT images. They were blinded to endoscopic results and presenting symptoms and disagreement on diagnosis was settled by consensus. For homogenous lesions, radiologists drew regions of interest (ROI) as big as possible to cover most part of the mass region, otherwise ROI were drawn on solid components of lesion, with caution to avoid peripheral necrotic and fat area. Parameters of spectral images were measured three times at three consecutive image levels to get credible mean values by each expert. To maintain consistency in position, shape, and size of ROIs between the three phases, all images of the triple-phases enhancement were loaded simultaneously into the workstation, and the ROIs were propagated by applying the copy-and-paste function. The area of ROIs ranged from 10 mm^2^ to 30 mm^2^ on the basis of mass size and shape. Iodine concentrations (IC) in the lesions were measured from the iodine-based material decomposition (MD) image and normalized against that in the aorta to derive normalized iodine concentrations (NIC = IC (in lesion)/IC (in aorta)). And the spectral attenuation curves were automatically generated by plotting the material attenuation against x-ray photon energy with GSI software by propagating the ROIs to all virtual monochromatic image sets with energies from 40 to 140 keV. Then slope of such curves (λ_HU_) was calculated as the CT attenuation difference at two energy levels (40 and 100 keV) divided by the energy difference (60 keV) from the spectral attenuation curve: λ_HU_ = (HU(40 keV)-HU(100 keV))/60.

### Statistical Analyses

The data obtained from the two radiologists were compared using the Bland-Altman analysis (MedCalc Software, Ostend, Belgium). The difference of IC, NIC and λ_HU_ in the three imaging phases between EGC and AGC was calculated by using the two-sample *t*-test. The comparison of the spectral CT parameters among the three Ki-67 antigen levels were performed by using the One-way analysis of variance and Dunnett *t*-test. The correlation between Ki-67 grade and IC, NIC and λ_HU_ values in the triple phases were further analyzed using the Spearman rank correlation analysis. The statistical analyses were performed by using the SPSS19.0 statistical software (IBM, New York, USA). *P* value of less than 0.05 or 0.01 was considered statistically significant.

## References

[1] Torre LA (2015). Global cancer statistics, 2012. CA. Cancer J. Clin..

[2] Ryun PS (2015). Management of gastric cancer: East vs West. Curr. Probl. Cancer..

[3] Japanese Gastric Cancer A (1998). Japanese Classification of Gastric Carcinoma − 2nd English Edition. Gastric Cancer..

[4] Bollschweiler E, Berlth F, Baltin C, Monig S, Holscher AH (2014). Treatment of early gastric cancer in the Western World. World J. Gastroenterol..

[5] Karimi P, Islami F, Anandasabapathy S, Freedman ND, Kamangar F (2014). Gastric cancer: descriptive epidemiology, risk factors, screening, and prevention. Cancer Epidemiol. Biomarkers Prev..

[6] Chen CY (2007). Gastric cancer: preoperative local staging with 3D multi-detector row CT–correlation with surgical and histopathologic results. Radiology..

[7] Grenacher L, Hansmann J (2007). Radiological imaging of the upper gastrointestinal tract. Part II. The stomach. Radiologe..

[8] Kim HJ (2005). Gastric cancer staging at multi-detector row CT gastrography: comparison of transverse and volumetric CT scanning. Radiology..

[9] Makino T (2011). Preoperative T staging of gastric cancer by multi-detector row computed tomography. Surgery..

[10] Filippone A (2004). Preoperative T and N staging of colorectal cancer: accuracy of contrast-enhanced multi-detector row CT colonography–initial experience. Radiology..

[11] Li C (2015). Computer-aided diagnosis for preoperative invasion depth of gastric cancer with dual-energy spectral CT imaging. Acad. Radiol..

[12] Pan Z (2013). Gastric cancer staging with dual energy spectral CT imaging. PLoS One..

[13] Yu L, Leng S, McCollough CH (2012). Dual-energy CT-based monochromatic imaging. A.J.R. Am. J. Roentgenol..

[14] Pang L (2011). Study of gemstone spectral imaging in diagnosis of gastric cancer. J. Surg. concepts Pract..

[15] Zhu X, Shen Y, Lin X, Liu Y, Chen K (2011). Use of CT Gemstone Spectral Imaging in the diagnosis of gastric pre-cancerous lesion and early gastric cancer. J. Diagn. Concepts Pract..

[16] Ross W, Hall PA (1995). Ki67: from antibody to molecule to understanding?. Clin. Mol. Pathol..

[17] Saricanbaz I (2014). Prognostic significance of expression of CD133 and Ki-67 in gastric cancer. Asian Pac. J. Cancer Prev..

[18] Zhao P, Li Y, Lu Y (2010). Aberrant expression of CD133 protein correlates with Ki-67 expression and is a prognostic marker in gastric adenocarcinoma. B.M.C. Cancer..

[19] Liu W (2011). Clinical significance of P53 and Ki67 expression in gastric cancer. W.C.J.D..

[20] Xin Y (1997). Study of the relationship between the expression of ki 67 antigen and the pathobiological behaviours of stomach cancer. Chin. J. Oncol..

[21] Böger C, Behrens HM, Röcken C (2016). Ki67–An unsuitable marker of gastric cancer prognosis unmasks intratumoral heterogeneity. J. Surg. Oncol..

[22] Jung KW (2013). Survival of korean adult cancer patients by stage at diagnosis, 2006-2010: national cancer registry study. Cancer Res. Treat..

[23] Nashimoto A (2013). Gastric cancer treated in 2002 in Japan: 2009 annual report of the JGCA nationwide registry. Gastric Cancer..

[24] Kim YH (2009). Staging of T3 and T4 gastric carcinoma with multidetector CT: added value of multiplanar reformations for prediction of adjacent organ invasion. Radiology..

[25] Johnson TR (2007). Material differentiation by dual energy CT: initial experience. Eur. Radiol..

[26] Fornaro J (2011). Dual- and multi-energy CT: approach to functional imaging. Insights Imaging..

[27] McCollough CH, Leng S, Yu L, Fletcher JG (2015). Dual- and Multi-Energy CT: Principles, Technical Approaches, and Clinical Applications. Radiology..

[28] Chen, K. *Principles And Clinical Applications Of Spectral* CT Imaging (ed. Chen, K.). 45–116 (Beijing, 2012).

[29] Chen CY, Wu DC, Kang WY, Hsu JS (2006). Staging of gastric cancer with 16-channel MDCT. Abdom Imaging..

[30] Lee JH (2000). Two-phase helical CT for detection of early gastric carcinoma: importance of the mucosal phase for analysis of the abnormal mucosal layer. J. Comput Assist Tomogr..

